# QTLs for bone mineral density of femurs and tibias in recombinant inbred strains derived from C57BL/6J and DBA/2J inbred strains

**DOI:** 10.1186/1471-2105-13-S12-A7

**Published:** 2012-07-31

**Authors:** Lishi Wang, Wenli Lu, Rachel Scheib, Yue Huang, XiaoYun Liu, Linda Myers, Lu Lu, Robert W Williams, Yan Jiao, Weikuan Gu

**Affiliations:** 1 Department of Orthopaedic Surgery - Campbell Clinic and Pathology, University of Tennessee Health Science Center, Memphis, TN 38163, USA; 2 Department of Medicine, University of Tennessee Health Science Center, Memphis, TN, 38163, USA; 3 Department of Anatomy and Neurobiology, University of Tennessee Health Science Center, Memphis, TN, 38163, USA

## Background

Quantitative trait loci (QTLs) for bone mineral density (BMD) are defined as regions of the genome that contain sequence variations that cause differences in either bone deposition or rates of resorption. In this study, we investigate QTLs for BMD of whole bones using femurs and tibiae from the BXD family of recombinant inbred (RI) strains derived by crossing C57BL/6J (B6) and DBA/2J (D2) inbred strains.

## Materials and methods

We studied femurs and tibiae from a total of 46 strains at 3 months-of-age. Bones were quantified using the PIXImus dual-energy X-ray absorptiometer (DXA) system. QTL mapping was carried out using simple and composite interval mapping in GeneNetwork (http://www.genenetwork.org). Candidate genes in QTL regions were ranked using PGMapper. SNP genotypes of candidate genes were verified directly using PCR amplification and sequencing.

## Results

Our data show:

1) A high correlation between BMD of the femur and tibia across the panel of BXD strains;

2) A high correlation between BMD of femur and tibia within sex and a moderate positive correlation between sexes;

3) A QTL on chromosome 15 for the BMD in femur and tibia in male mice located in a 10 Mb region between 42 and 52 Mb;

4) A total of 48 transcripts within the Chr 15 QTL or which three are particularly attractive candidate genes—*Trps1*, *Ext1*, and *Slc30a8*.

## Conclusions

We have identified QTLs for BMD using a set of 46 BXD RI strains. Further investigation of the three candidate genes located in this QTL on chromosome 15 is essential. Despite limitations, PIXImus is a valuable tool for studying BMD and skeletal development of small animals.

